# Effects of mis-alignment between dispersal traits and landscape structure on dispersal success in fragmented landscapes

**DOI:** 10.1098/rsos.181702

**Published:** 2019-01-16

**Authors:** Justine L. Atkins, George L. W. Perry, Todd E. Dennis

**Affiliations:** 1 Department of Ecology and Evolutionary Biology, Princeton University, 106A Guyot Hall, Princeton, NJ 08544-2016, USA; 2 School of Environment, University of Auckland, Private Bag 92019, Auckland 1142, New Zealand; 3 Department of Biology, Fiji National University, PO Box 5529, Natabua, Lautoka, Republic of Fiji Islands

**Keywords:** agent-based model, dispersal, biological trait, trade-off, spatially explicit simulation model, virtual ecology

## Abstract

Dispersal is fundamental to population dynamics and hence extinction risk. The dispersal success of animals depends on the biophysical structure of their environments and their biological traits; however, comparatively little is known about how evolutionary trade-offs among suites of biological traits affect dispersal potential. We developed a spatially explicit agent-based simulation model to evaluate the influence of trade-offs among a suite of biological traits on the dispersal success of vagile animals in fragmented landscapes. We specifically chose traits known to influence dispersal success: speed of movement, perceptual range, risk of predation, need to forage during dispersal, and amount of suitable habitat required for successful settlement in a patch. Using the metric of relative dispersal success rate, we assessed how the costs and benefits of evolutionary investment in these biological traits varied with landscape structure. In heterogeneous environments with low habitat availability and scattered habitat patches, individuals with more equal allocation across the trait spectrum dispersed most successfully. Our analyses suggest that the dispersal success of animals in heterogeneous environments is highly dependent on hierarchical interactions between trait trade-offs and the geometric configurations of the habitat patches in the landscapes through which they disperse. In an applied sense, our results indicate potential for ecological mis-alignment between species' evolved suites of dispersal-related traits and altered environmental conditions as a result of rapid global change. In many cases identifying the processes that shape patterns of animal dispersal, and the consequences of abiotic changes for these processes, will require consideration of complex relationships among a range of organism-specific and environmental factors.

## 1. Introduction

Dispersal between interacting but spatially segregated populations of conspecific animals is essential for the maintenance of gene flow, recolonization following local extirpation, population persistence, response to environmental change, and, ultimately, reduction of extinction risk [1–5]. Understanding the factors that influence dispersal therefore is critical for the maintenance of biodiversity, especially as large previously intact natural habitats become increasingly degraded and fragmented by human activity [6–9]. The dispersal success of animals is influenced by a broad range of abiotic and biotic factors. Numerous studies have shown that both landscape composition (what and how much is there?) and structure (how is it distributed in space and time?) are key determinants of the movements and fates of dispersing animals [3,6,10–13]. Much also is now known about the importance of non-random (targeted) dispersal and the role that biological traits, both at the species and inter-individual-level, play in determining dispersal success [14–19]. These phenotypic attributes interact with the environment in various ways to regulate dispersal processes [20,21]. Fundamental biological traits known to affect animal dispersal include: movement capacity; energetic requirements; behavioural plasticity; perceptive and cognitive abilities; foraging strategies; philopatric tendencies; and mating systems [21,22].

The critical importance of biological traits to animal dispersal implies that there should be strong selection pressure to evolve dispersal strategies that optimize key attributes in relation to particular life histories and environments [23,24]. As with any biological trait, there are physiological constraints and trade-offs that delimit the dispersal strategies that species use; for example, contrasting needs for rapid maturation and reproduction may result in reduced growth of limbs which subsequently restricts dispersal capacity [23,25,26]. Extant dispersal strategies also depend on the environmental contexts in which they developed [27]; theoretical work has demonstrated that dispersal strategies evolve in response to changes in habitat availability, noise or heterogeneity (through both space and time) and landscape pattern [28,29]. Dispersal rates that are stable over evolutionary time are also determined by the specific environment and biological traits of species or their sub-populations [30].

Given the current rapid pace of anthropogenically-induced environmental change, there is increased risk that the dispersal traits of species, theoretically optimized for performance in their specific ecological niches, may become mis-aligned with their current environmental conditions [31,32]. An increase in habitat fragmentation, in particular, can have major effects on dispersal behaviour. Fragmentation increases the distances between suitable habitat patches and often reduces the size and/or quality of these patches, isolating populations and intensifying the effects of processes such as demographic stochasticity and inbreeding [7]. Theoretical modelling of dispersal evolution and success (i.e. the event of an organism moving from one habitat patch to another without suffering mortality) suggests that the potential outcomes of increased fragmentation for dispersal are complex, but that dispersal propensity (i.e. the tendency to leave a patch and initiate a dispersal event) generally, will be affected in most cases. On the one hand, fragmentation may select for reduced dispersal rates (due to greater distances among patches and higher risk of mortality or costs when moving through the matrix) [28,33,34]. On the other hand, by reducing the size and/or quality of the habitat patches, fragmentation may promote long-distance dispersal necessitated by sub-optimal conditions in the natal environment [27,30,35]. In a recent review, Cote *et al*. [7] highlighted the wide-ranging effects that habitat fragmentation could have on dispersal syndromes—suites of biological traits that covary with dispersal decisions, and that vary among individuals in the same population [36]. Importantly, the consequences of fragmentation (e.g. changes to inter-patch distance, predation risk, matrix quality, competition) seem likely to select for particular dispersal syndromes over others, and increased variability in environmental conditions among habitat patches may, in turn, increase spatial structuring of populations, local adaptation and ultimately promote disruptive selection on species' dispersal strategies [35,37–40].

Evaluating the costs and benefits of evolutionary investment in different traits in environments with a range of landscape structures will improve our understanding of how species may respond to increasing environmental perturbation, specifically fragmentation. Dispersal traits that are likely to shift include emigration and immigration probabilities, the distance moved during dispersal, and transience success [7]. These changes, in turn, will select for associated shifts in other phenotypic traits such as body size, characteristics related to mobility and locomotive speed, and anti-predator strategies [7]. Empirically characterizing the relative importance of these myriad traits on dispersal success in landscapes with different levels of fragmentation is challenging, because it requires systematically assessing the movements, behaviour and fate of dispersing animals, typically over large spatial and temporal extents [11,41–43]. Research using EMS (Experimental Model Systems) has addressed questions about the effects of perceptual range, matrix composition and connectivity pathways on movement across landscapes [44–46]. Nevertheless, the potential combinations of traits and environmental conditions to be considered present such a vast parameter space that more theoretical approaches must be considered to: (i) provide support for observational patterns and (ii) identify which biological traits and/or dispersal syndromes will be selected for under increasing fragmentation. Findings from such theoretical work could also then be fed back into EMS for more targeted experimentation.

Spatially explicit agent-based models (SEABM) have illuminated some overarching principles describing the effects of biological traits and their interactions with landscape context for various movement behaviours including the evolution of dispersal [47]. For example, Buchmann *et al*. [48–50] developed an SEABM of home-range formation that considers the allometric relationships evident in habitat-selection processes, and explores the consequences of such relationships for community assembly and response to habitat loss and fragmentation. The RangeShifter software uses an SEIBM to link individual dispersal behaviour and population dynamics, facilitating the study of species' responses to rapid environmental change [51].

Here, we use an SEABM to examine the relative importance of a variety of individual-level biological traits and evaluate how these traits influence dispersal success in landscapes with different levels of fragmentation. We focus on the transient stage of dispersal (i.e. when the organism has departed the origin patch and before it reaches the settlement patch) as this is the stage for which the consequences of fragmentation (i.e. increased costs) are likely to be most immediately influential [7,23]. We assess the role of landscape context in determining which combination of traits (in terms of relative evolutionary investment) are the most important predictors of dispersal success, which we define as the event of an organism moving from one habitat patch to another without suffering mortality. With this model design, we aim to provide theoretical support for trends identified from observational data, identifying which biological traits may be selected for under increasing fragmentation and the potential for disruptive selection on alternate dispersal syndromes. The traits we examine may vary both within- and between-species; thus, the results of our simulations can be used to identify both species that may have a selective advantage under increasing habitat fragmentation, and particular phenotypes, or dispersal syndromes, within a species that may be more successful in these scenarios. We assessed five biological traits that are likely to influence dispersal success in fragmented landscapes (see [7,52]), and therefore likely to be under selection pressure in changing environments: movement speed, perceptual range, mortality rate during dispersal (as a proxy for predation risk/investment in anti-predator strategies), minimum habitat area requirements, and tendency to forage during dispersal. Individuals were allocated a limited ‘evolutionary investment budget’ and forced to trade-off investment among these traits (see [53,54]). We then evaluated how differential investment in each biological trait affected dispersal success, both independently and through direct interactions with landscape characteristics. We hypothesized that increased fragmentation would result in lower dispersal success overall, but that dispersal strategies that emphasized fast speed of movement between patches would be the most successful, especially as fragmentation increased [55]. Our simulation model was developed to be deliberately generic so that our findings may be applicable to many systems in which individuals routinely disperse among habitat patches and fragmentation of important habitats may occur.

## 2. Methods

### 2.1. Model description

We implemented our simulation model in NetLogo 5.0.4 [56] and represented the dispersal of individuals (animals) between discrete patches of suitable habitat in variously fragmented landscapes. Our model runs were executed on a grid of 100 × 100 cells. Simulated landscapes were mosaics of discrete habitat patches embedded in a non-habitat matrix characterized by varying levels of ‘quality’ (representing, for example, available food resources). In this paper our use of the term ‘landscape’ does not imply our findings are relevant only to terrestrial environments; the processes we consider herein are equally important in other patchy environments, such as marine benthic ecosystems (‘seascapes’). Electronic supplementary material 1 provides a detailed description of our model in the standard Overview, Design concepts and Details (ODD) format of [57].

We determined the spatial distribution of suitable habitat patches within model landscapes using two parameters: habitat amount (‘HABAMT’ [0–100%]) and habitat aggregation (‘HABAGG’ [0–1]; table 1). HABAMT set the surface area (%) of suitable habitat in landscapes, while HABAGG defined the extent of spatial clustering of habitat patches. The degree to which suitable habitat was clustered determined both the mean size (number of grid cells) of habitat patches and the mean distance between patches. To simulate different levels of habitat loss and fragmentation, we varied HABAMT from 5% to 50%, at 5% increments, while HABAGG was varied from 0.0 to 0.75 at 0.25 intervals (for details, see electronic supplementary material 1).

**Table 1.** Parameters/variables and their values for the dispersal-simulation model.

parameter/variable	value/range of values
landscape	
total landscape area	100 × 100 cells
representative area of each grid cell	50 × 50 m
quality value of a habitat patch	0–1.0
quality percentile for ‘forage’ patches	highest 15%
habitat amount (HABAMT; as % of total landscape)	5–50%
habitat aggregation (HABAGG; aggregation or spatial ‘clustering’ of individual patches)	0–0.75
individual traits	
movement speed	1–5 cells per-step
mortality probability (per-step)	0.0001, 0.001, 0.005, 0.01, 0.015
perceptual range	1–5 cells
minimum patch area (MINAREA)	1–50 cells
foraging tendency (probability)	0.0–0.5
maximum turning angle of correlated random walk	90°
angle of perceptual range	180°
energy gained from foraging (per-step)	1 step
foraging speed	0.1 cells per-step
population	
initial individuals initialized	2500
temporal scale	
maximum number of steps (stopping point)	250
length of each time-step	approx. 1 min (implied)

Using our model, we assessed the effects of the relative importance of different biological traits on dispersal success in varying landscape structures. We used local averaging [58] to generate heterogeneous landscape matrices (i.e. the cells between habitat patches) that varied in the quality of food resources. This process involved seeding all cells in a landscape matrix with a random uniform deviate (U ∼ [0,1]), and then applying a local-averaging function at the desired intensity over each cell's eight neighbours (i.e. a Moore neighbourhood). The total amount of food resources available in a landscape remained constant during this procedure, but the spatial distribution of resources was varied. Matrix cells that had ‘forage-quality’ values (see below) falling in the upper 15% of the distribution were deemed to be ‘foraging-patches’, in which individuals could find food resources during a dispersal event.

We assessed the importance of different biological traits to dispersal success by assigning various traits to individuals (e.g. movement speed and perceptual range), and then manipulating the values of those traits during simulation experiments (see table 2 for attribute descriptions). Dispersal movements between habitat patches followed a correlated random walk ([59]; maximum turning angle 90°), with individuals transiting from their starting habitat patches through the landscape matrix until a suitable patch was detected within a perceptual ‘window’, at which point the suitable habitat patch was then directly approached. If during a dispersal event an individual encountered ‘foraging’ cells in the landscape matrix, the model agent was programmed to switch into ‘foraging mode’ at a probability determined by its inherent biological ‘foraging tendency’. In all model realizations, the spatial configuration of habitat patches within the landscape influenced whether an individual dispersed successfully, because the number of model steps (time) available to find a habitat patch was limited (at 250 steps; preliminary analysis indicated this was sufficient for model agents to achieve dispersal success if they were going to be successful, see electronic supplementary material 1).

**Table 2.** State variables for individual dispersing individuals in the simulation model.

state variable	description
start habitat	habitat patch where an individual is randomly initialized
current habitat	habitat patch where an individual is currently located
total steps	cumulative number of model steps taken by an individual
movement mode	the behavioural mode in which an individual moves—either foraging or dispersing
biological traits	
movement speed	speed at which an individual moves, measured in NetLogo cells per model step
perceptual range	distance over which an individual can ‘see’ the landscape, measured in NetLogo cells
background mortality rate	probability an individual dies during dispersal, per model step (proxy for predation risk during dispersal)
minimum patch area (MINAREA)	minimum area of a habitat patch required for settlement, measured in NetLogo cells (proxy for energetic constraints that require a minimum area for subsistence)
foraging tendency (probability)	probability an individual forages when entering a foraging patch (proxy for energetic constraints that require foraging during dispersal)

### 2.2. Model realizations

**Figure 1.**
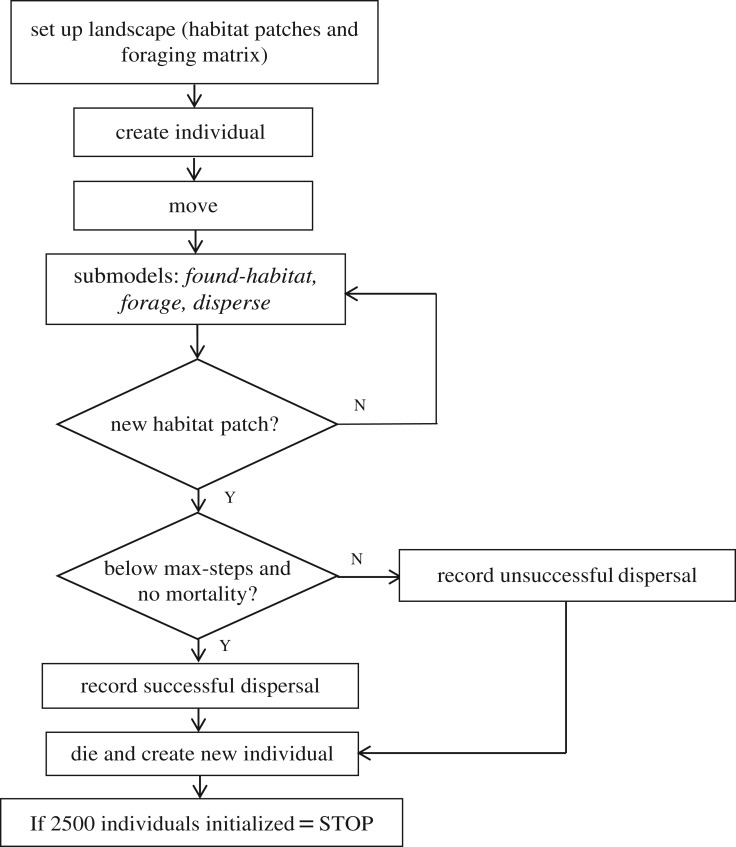
Flowchart of the model schedule and operation. Diamond-shaped boxes represent decisions made by the model individuals (dispersing individuals) for which the outcome was either: Y (yes) or N (no). First, the landscape was generated, then an individual was initialized and progressed through the sub-models; finally, dispersal attempts were recorded as successes or failures. This procedure was repeated until 2500 individuals had attempted to disperse, comprising a single model run. ‘Max-steps’ represents the maximum number of model steps permitted for the dispersal attempt of a single model individual (set at 250).

Figure 1 provides a schematic of the model scheduling. After generating the landscape, we placed individual model agents, one at a time (there were no interactions with other individuals), at the edge of a randomly selected habitat patch. Individuals then moved through the landscape until they either dispersed successfully (i.e. settled in a habitat patch different from that they started in) or died. Death could occur either because an individual suffered background mortality (at a fixed probability per model step), or because the maximum number (250) of permitted model steps was exceeded (table 1; see electronic supplementary material 1 for a description of how stopping points were calculated). For all model scenarios we repeated this procedure until 2500 individuals had attempted to disperse. We defined dispersal success for each model realization as the *proportion* of individuals that successfully moved from their original habitat patch to another patch without dying (see electronic supplementary material 1).

### 2.3. Simulation experiments and analysis

We examined relationships between dispersal success, trait-parameter values and the influence of different levels of investment in biological traits on dispersal success by conducting a series of simulation experiments. First, to assess whether our model was ‘fit-for-purpose’, we varied HABAMT and the level of HABAGG in the model landscapes (see table 1 for range values), as well as the parameter values of each biological trait to assess how each trait influenced dispersal success independently. For each trait, we conducted 100 simulations for 40 combinations of the environmental parameters (i.e. 10 levels of HABAMT and four levels of HABAGG) and five parameter values of each biological trait. During these model runs, only one trait was varied at a time, with all other parameters fixed at baseline values (centres of their range values; see table 1). Overall, the modelling procedure resulted in 21 × 10^4^ runs of the model for each biological trait (for five biological traits, resulting in 1.05 × 10^6^ simulations) and 200 mean estimates of dispersal success from all possible combinations of 40 environmental scenarios and five trait values.

We then explored the full model parameter space using Latin hypercube sampling (‘LHS’; [60]); in this way, we conducted 1 × 10^4^ unique simulations (see also electronic supplementary material 2 for a description of LHS). We did not replicate individual parameter combinations but instead tried to cover as much of the parameter space as possible, as recommended in [61]. We analysed the outcomes of simulations using boosted regression trees (BRT; [62,63]), which provided a means of exploring relationships between predictor variables (biological traits and landscape factors) and the dependent variable ‘dispersal success’. We measured the relative contribution of each predictor variable in terms of the extent of its contribution to accurate prediction of dispersal success. In the BRT analysis, we used the following hyper-parameter settings: learning rate = 0.075 for assessment of all model parameters and 0.025 for assessment of only intrinsic-trait parameters; distribution family = ‘Gaussian’; tree-complexity = 5; and bag fraction = 0.75. Because preliminary analysis showed that our results were clearly dominated by landscape factors, we re-sampled the model-parameter space, and repeated the BRT analysis using only the biological-trait parameters, with values for HABAMT and HABAGG held constant at the mid-points of their ranges.

In a second set of experiments, we assigned each model agent a limited ‘trait-investment budget’. We randomly allocated individual agents proportions of this budget to each trait, which essentially determined relative investment in and capabilities for movement speed, perceptual range, mortality probability, minimum patch area required for settlement (MINAREA) and foraging tendency. To allocate trait-investment budgets, we employed a ‘broken-stick’ random-number generation method [64] (see also electronic supplementary material 2). Unlike the parameter-space evaluation described for the first set of experiments, our trait-investment analysis did not permit individuals to optimize multiple traits concurrently—rather, investment in one biological trait equated to less investment in the other traits. This approach allowed us to examine how investment in specific traits influenced dispersal success. We then assessed the role of landscape context in determining the combination of traits (in terms of relative importance) that best predicted dispersal success by varying HABAMT and HABAGG. We repeated this trait-specialization procedure 1000 times in landscapes containing 5%, 10%, 15% and 20% suitable habitat (HABAMT), at two levels of HABAGG (0.1 and 0.4; i.e. eight different landscapes in total). We assigned each model replicate different parameter values for biological traits, representing a unique combination of relative investment among biological traits. We characterized the degree of investment in biological traits using the Shannon diversity index [65]. This index was higher when the ‘budget’ was spread more evenly among the five traits and lowest when one or two traits were assigned the majority of the resources. We then used this metric to assess the advantages of each trait and, importantly, investment in different combinations of these traits, for dispersal success.

We compared the specific allocation of resources to each of the biological traits with dispersal success both graphically and using quantile regression analysis (see [66,67]) to quantify the influence on the dispersal success of individuals with different trait combinations—i.e. high or low resource allocation to certain traits. We made this comparison for each environmental scenario (i.e. each combination of HABAMT and HABAGG), allowing us to examine trade-off dynamics under changing external conditions and assessment of how the advantages of different trade-offs are altered when these conditions change.

Our analyses focused on the model's behaviour and dynamics via effect sizes of dispersal success as a function of biological traits and landscape structure. This approach is preferred over formal frequentist tests for assessing large simulated datasets, because with such data even small differences in model outcomes can be significant due to high statistical power [68,69]. Thus, we adopted an ‘evaluative’ perspective, in which the relative influences (effect sizes) and relationships among parameter values were assessed in terms of their ability to influence dispersal success.

## 3. Results

**Figure 2.**
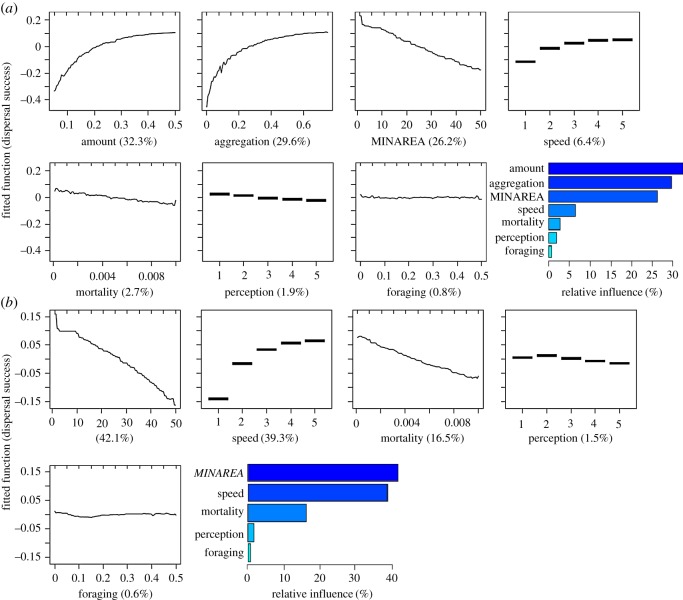
Relative influence of model parameters on dispersal success as derived from boosted-regression-tree (BRT) analyses. (*a*) Both biological traits and environmental factors were varied. ‘Amount’ (HABAMT) and ‘aggregation’ (HABAGG) are landscape parameters of amount of available habitat and aggregation among habitat patches. Individual trait parameters are (upper left to lower right): habitat (minimum habitat-area (MINAREA)—the number of cells required for a patch to be suitable habitat); movement speed (NetLogo cells moved per model step); mortality probability (per step); perceptual range (in NetLogo cells); and the probability of foraging. The ‘fitted value’ is the value of dispersal success derived from the fitted BRT model. The column plot in the lower right corner shows the relative influence of each predictor variable on dispersal success in the BRT model. Lines on the upper *x*-axis indicate the marginal distribution of predictors across the sample. (*b*) Landscape parameters were held constant at median values of their ranges and each biological trait parameter was varied.

Boosted-regression-tree (BRT) analysis of the full model-parameter space (table 1) indicated that the amount and spatial aggregation (i.e. ‘clustering’) of habitat patches were, overall, the two most important model parameters determining dispersal success (relative contributions of 32.3% and 29.6%, respectively) (figure 2*a*). Of the biological traits we assessed, movement speed in all cases was positively associated with dispersal success, and MINAREA and background mortality rate were negatively correlated with dispersal success. Perceptual range and foraging tendency showed little correlation with dispersal success, and the contributions of these traits to dispersal success were less than 10% (figure 2*a*). These multivariate assessments were corroborated by sensitivity analysis, in which each biological trait was varied individually for landscapes that differed in values of HABAMT and HABAGG. An exception to corroboration with sensitivity analysis was perceptual range, which showed a slight positive correlation with dispersal success (see electronic supplementary material 3).

For the BRT models that evaluated only biological traits, the rank order of importance of traits was the same as in the full model (figure 2*b*). MINAREA, again, was negatively correlated with dispersal success, but had more influence than in the full model (relative contributions = 42.1% versus 26.2%, respectively). Similarly, movement speed had a much stronger positive, and background mortality rate a stronger negative, association with dispersal success (relative contributions of 39.3% and 16.5%, respectively); perceptual range and foraging tendency were again comparatively unimportant (contributions of less than 2%).

**Figure 3.**
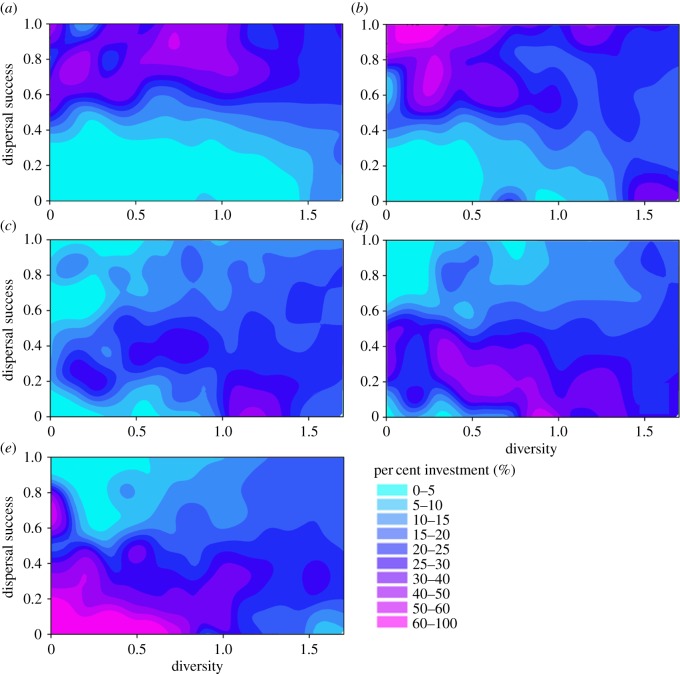
Variation in dispersal success across a fragmented landscape along a continuum of relative investment in biological traits. Each panel shows the dispersal success of model runs with different Shannon diversity indices resulting from unique trade-offs of biological traits, in a landscape of 20% suitable habitat (HABAMT) and with habitat aggregation = 0.4 (HABAGG). Filled contour surfaces depict the degree of specialization in (*a*) movement speed, (*b*) mortality probability, (*c*) perceptual range, (*d*) foraging tendency and (*e*) minimum patch area (MINAREA).

In the second set of experiments, we examined how dispersal success was influenced by relative investment in each biological trait. When the amount of habitat was not limiting (HABAMT ≥ 20%), we found a clear advantage to investing in just one or two biological traits (figure 3). These traits were movement speed and all unspecified adaptations that reduced the predation-related mortality rate (which were not explicitly incorporated into our model but are exemplified by crypsis, aposematism, chemical defences, deimatic behaviour, etc.). For these two traits, dispersal success was *ca* 20% higher when investment in each was at least 30%, with a Shannon index < 1 (the maximum value the Shannon index can take for five traits is 1.609; figure 3*a*,*b*). Perceptual range, as represented in the model, was not as important for dispersal success (figure 3*c*); investment in foraging tendency (interpreted, for example, as the need to forage during dispersal due to high metabolic rate, low capacity for energy storage) and reduction in MINAREA requirements decreased dispersal success below mean values. This outcome was a consequence of the concomitant loss of investment in the more valuable traits of movement speed and predation-avoidance adaptations (figure 3*d*,*e*).

**Figure 4.**
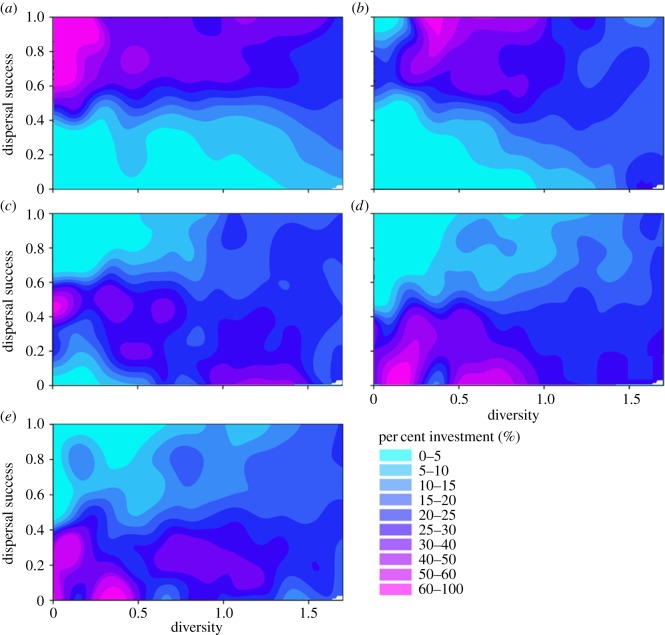
Variation in dispersal success across a fragmented landscape along a continuum of relative investment in biological traits. Each panel shows the dispersal success of model runs with different Shannon diversity indices resulting from unique trade-offs of biological traits, in a landscape of 5% suitable habitat (HABAMT) and with 0.4 degree of habitat aggregation (HABAGG). Filled contour surfaces depict the degree of specialization in (*a*) movement speed, (*b*) mortality probability, (*c*) perceptual range, (*d*) foraging tendency and (*e*) minimum patch area (MINAREA).

The clear advantages to dispersal success of increased movement speed and reduced predation-related mortality increased as the amount of suitable habitat in landscapes decreased to 5%. In particular, when HABAMT was limiting, individuals that invested mostly in increased movement speed (60–100%, with a Shannon Weaver index < 0.5) experienced consistently high dispersal success (0.6–1.0), while individuals with more moderate investment in this trait (15–60%) showed more variable dispersal success (0.2–1.0) (figure 4). This trend was also evident for increased investment in mortality-avoidance traits (figure 4), with the exception of high dispersal success eventuating for a small number of individuals that had 0–5% investment in this trait (these were likely the same individuals that had close to 100% investment in movement speed, which subsequently experienced the greatest dispersal success).

**Figure 5.**
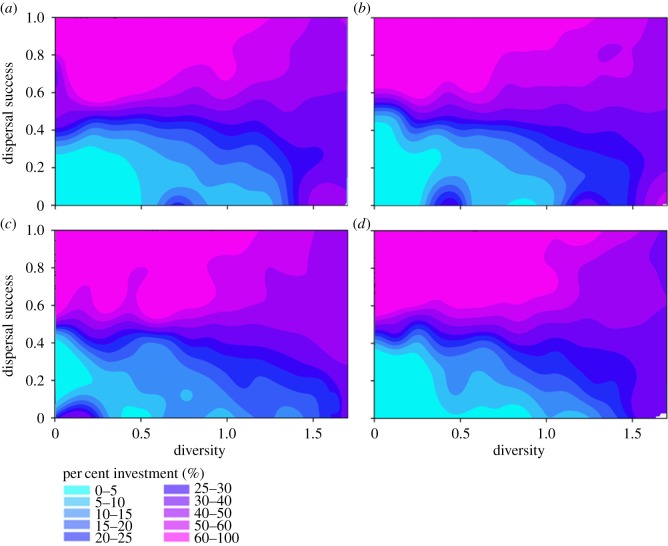
Variation in dispersal success due to different trade-offs of relative investment in speed and mortality-avoidance traits. Trade-offs between the two traits are expressed as Shannon diversity indices, and the relationships is assessed in a fragmented landscape with 20%, 15%, 10% and 5% proportion (area) of suitable habitat and 0.4 degree of habitat aggregation (HABAGG). The size of the circles and colour scale of the contour surface both depict the relative degree of evolutionary investment in movement speed and mortality-avoidance traits.

Given the clear importance of movement speed and anti-predation traits to dispersal success, we then combined the relative investment in both into a single score and examined the effects of this joint-investment level. In landscapes with only 5% suitable habitat, agents with above-average investment in high movement speed and low predation-related mortality dispersed more successfully than other individuals (mean dispersal success was *ca* 80% and 55%, respectively; figure 5). A similar pattern, although weaker, was evident for landscapes with 20% suitable habitat (figure 5). In landscapes with intermediate amounts of suitable habitat (10% and 15%), the dispersal success of individuals with above-average investment in movement speed and predation-avoidance adaptations was more variable (ranging from 10% to nearly 100%; figure 5).

**Figure 6.**
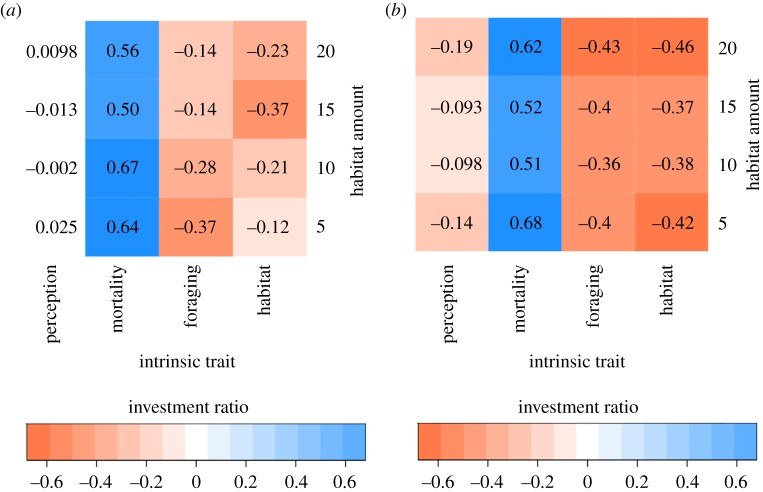
Slopes of the quantile regression for the 25th percentile of trade-off scenarios with the lowest dispersal success, for each biological trait, in landscapes of varying habitat amount with degree of aggregation among habitat patches set at 0.4 (*a*) and 0.1 (*b*). Slopes represent the proportional increase in dispersal success for the degree of relative investment in a particular trait. As movement speed consistently showed the largest payoff for dispersal success, slopes from the quantile regression for the other biological traits were converted into ratios of the payoff in dispersal success standardized with respect to that of movement speed. ‘Investment’-payoff ratios were calculated for four different levels of the proportion (amount) of suitable habitat in the environment. For example, in a landscape with 10% of the total area that was suitable habitat, the probability of mortality per time step had a payoff that was approximately half that of movement speed (ratios of 1 : 1 means that payoffs are equal to that of speed). ‘Perception’ indicates the perceptual range of the dispersing agent, ‘mortality’ is the per-step probability of mortality during dispersal, ‘foraging’ is probability of foraging during dispersal and ‘habitat’ is the minimum habitat-area requirement of individuals.

The landscape-dependent advantages for dispersal success of a combination of high movement speed and low predation-related mortality are further supported by quantile regressions (figure 6*a*). Quantile regression ratios showed a bell-shaped pattern in relation to the proportion (‘amount’) of habitat in the landscape (‘HABAMT’; figure 6*a*). Landscapes with the highest (20%) and lowest (5%) amounts of suitable habitat corresponded with the highest investment ratios (whether positive or negative) for each biological trait, relative to speed. In particular, for these two environments, the payoff for investment in predation-avoidance traits was between 62% and 68% of the payoff for investment in movement speed, compared to 51–52% in other environments. This finding suggests that the advantage of investing in speed was greatest in intermediate environments (10% and 15% suitable habitat), while in more extreme environments, it was more advantageous to invest equally in speed and predation-avoidance traits. Joint investment in movement speed and predation-avoidance traits was the most advantageous trait combination in environments with very low proportions of suitable habitat (below 5%). Overall, in landscapes with limited amounts of suitable habitat (and thereby smaller habitat patches and higher mean distance among patches), it was advantageous for dispersing individuals to invest in both higher movement speed and increased ability to avoid predation, effectively adopting a ‘bet-hedging’ strategy directed towards the two most important biological determinants of dispersal success.

In the results described above, the degree of habitat aggregation (‘HABAGG’) was held constant at a value of 0.4. We also examined the effects of biological trait trade-offs on dispersal success with varying amounts of habitat (‘HABAMT’) in landscapes with an aggregation value of 0.1 (i.e. increased fragmentation of habitat patches). The results of these simulations are similar to those in landscapes having a higher degree of aggregation, showing that when HABAMT is not limiting, individuals that invest in just one biological trait, speed of movement, have greater dispersal success, and *vice versa* (figure 6*b*). However, the lower aggregation among habitat patches resulted in the greatest payoff for investment in anti-predator traits occurring in landscapes with lower amounts of suitable habitat (landscapes with 5% and 10% habitat had *ca* 10% greater payoff than landscapes with 15% and 20% habitat amount; figure 6*b*). This result contrasts with what occurred in landscapes with higher habitat aggregation (discussed above), in which the greatest payoff eventuated in landscapes with 5% and 20% habitat (figure 6*a*). Finally, while high investment in biological traits other than speed of movement or predator-avoidance attributes reduced dispersal success, the cost of investment in these traits was comparatively lower in highly fragmented landscapes (comparing the negative payoffs for ‘Perception’, ‘Habitat’ and ‘Speed’ between figure 6*a* and *b*).

## 4. Discussion

We used a spatially explicit agent-based model to explore the relative importance of different biological traits for the dispersal success of free-ranging animals in fragmented, heterogeneous landscapes. Our results provide evidence for potential mis-alignment between dispersal-related biological traits and changing environmental conditions: only certain biological traits or combinations of traits result in sufficient rates of dispersal success, and these vary across fragmentation scenarios. Collectively, our analyses also indicate that landscape structure and the specific nature of the habitat fragmentation play an important role in determining the combination of traits that determine dispersal success.

Unsurprisingly, speed of movement was the most important trait for dispersal success in all landscapes, and particularly in landscapes containing intermediate amounts of habitat (10% and 15%). Therefore, individuals or species that can disperse rapidly between habitat patches likely will be less affected by fragmentation. Likewise, traits that reduce the risk of mortality during dispersal (i.e. anti-predator strategies) should be sensitive to increasing selection pressure. Contrastingly, species or individuals that are energetically constrained to settle in large habitat patches or to forage frequently during dispersal most probably will experience lower dispersal success in increasingly fragmented environments.

Our findings are consistent with previous studies on the evolution of dispersal strategies. Balancing the energetic costs of movement, time allocation and predation risks associated with movement through non-habitat areas are three of the major selection pressures on the evolution of dispersal strategies [23,70]. In field studies, individuals dispersing between more isolated sub-populations suffer higher predation rates, as they experience higher exposure to depredation [71]. Modelling studies have shown that optimal search-strategies are dependent on the predation rate imposed on individuals [72]. The results of our study confirm that predation-caused mortality is a determinant of dispersal success, and again show that the duration of dispersal events is central to the effectiveness of any particular strategy. Faster-moving organisms reduce the duration of dispersal events, and thereby experience lower exposure to all the different risks inherent in dispersal.

Our results also provide evidence that the importance of a given trait for dispersal success varies with landscape context. For example, while speed of movement is clearly an important component of dispersal success, in some landscapes, it is more advantageous to have more equitable investment in improving speed of movement and in adaptations that reduce per-step mortality rate (e.g. anti-predator strategies). Recent work has suggested that relationships between mobility and extinction risk also depend on a species' historic landscape structure and the subsequent degree of fragmentation [55]. Further, we find that these interactions cannot be accurately predicted solely by habitat availability. A comprehensive understanding of the outcomes for dispersal success of differential investment in each biological trait also requires information about how the environment is fragmented (i.e. need a combination of both habitat amount and aggregation values) [73]. We also found a reduced cost (and therefore a potential advantage) to investment in other biological traits such as perceptual range when aggregation among habitat patches was decreased, which has been observed empirically [74,75].

There are many predictions, together with some empirical evidence, regarding how habitat fragmentation, specifically the extent of habitat clustering, affects dispersal success and dispersal-related biological traits [7,73,76]. Focusing on the transfer stage of dispersal, our results corroborate the theory that most fragmentation scenarios are likely to significantly disrupt dispersal. Importantly, we provide support for the idea that such disruption of the dispersal process may select for alternative dispersal syndromes. If there is local environmental variation in the extent or nature of fragmentation, our model suggests that different dispersal traits will be advantaged (e.g. fast, long-distance dispersers versus a phenotype combining moderate speed with survival-enhancing traits or improved detection skills and use of information during dispersal). Cote *et al*. [7] proposed that such disruptive selection could enhance within-population variation and potentially facilitate local adaptation and the spatial structuring of populations based on their dispersal tendencies. If fragmentation continues to increase, some of these sub-populations thrive while others struggle to persist, ultimately resulting in a loss of important phenotypic variation, both at the species and within-species levels.

We caution that generic assumptions regarding the population responses of species to landscape fragmentation may lead to erroneous inferences regarding sensitivity to extinction risk due to loss of key habitats. Failure to disperse successfully increases the physical and genetic isolation of population networks, which ultimately elevates extinction risk [71,77,78]. The fate of dispersing animals is believed to be determined predominantly by landscape composition and configuration [79–81]. Our simulation experiments suggest, however, that segregation of sub-populations of animals inhabiting heterogeneous landscapes can develop as a consequence of the idiosyncratic movement behaviour of individuals that emerges from interactions between their unique syndrome of biological traits and environmental structure (see also [82]). Biological attributes, whether considered independently or in relation to environmental context, can render animal populations functionally segregated irrespective of their geographical proximity. Likewise, position along a continuum of dispersal-related biological traits may differentially influence dispersal success and thereby affect population persistence [83–86]. If so, empirical studies of animal dispersal likely will benefit from inclusion of species- or individual-specific attributes [21].

Models, by definition, are simplified representations of more complex systems [58,87], and so inferences drawn from models depend largely on how adequately they represent the phenomena of interest. Several important components of the dispersal process were not represented in our model; it is important to consider how these assumptions may have impacted the conclusions drawn from our study. Most obviously, density-dependent settlement was not taken into account, and this likely contributed to the relatively high rates of dispersal success observed in our simulations. Density-dependent habitat selection would likely reduce dispersal success by reducing ‘available’ settlement sites.

Our analyses also show a cost to dispersal success from increased investment in perceptual range or foraging tendency in some scenarios. These results were unexpected based on established literature (see [88]) and can be explained by two key simplifications that were necessary for development and analysis of a tractable model. First, perception and foraging tendency are tightly coupled with energetic dynamics, and increased perceptual range is associated with higher energetic costs [89]. Incorporating the energetic cost of increased investment in sensory systems, such as perceptual range, would improve the representation of this trait. In the case of foraging tendency, optimal foraging theory asserts that the timing and location of foraging bouts depend on the balance between the costs and benefits of different food resources and locations [90,91]. Our model represented this balance by having individuals forage only in the top 15% of high-quality matrix patches, gaining more search time through an increased number of steps and reducing net movement distances by foraging at one-tenth of dispersal speed. This is, of course, a simplistic representation of the complex energetic dynamics of foraging processes. Detailed agent-based models of foraging behaviour exist but are well beyond the scope of dispersal models intended to evaluate the importance of multiple biological traits (e.g. [48,92,93]). Second, the broken stick methodology we used for trait investment did not permit individuals to simultaneously optimize multiple traits—with a set ‘budget’, investment in one biological trait necessarily led to lower investment in other traits. While this approach allowed us to address our central aim of identifying the individual traits of greatest importance to dispersal success under different landscape scenarios, it did prevent exploration of potential positive correlations among our chosen biological traits (e.g. between speed of movement and perceptual range). Such an analysis would be a natural extension of our model and a necessary bridge between the theoretical ideas that we explored in this study and observational data on changing dispersal dynamics in species of different body sizes, for example.

A key benefit of SEABMs is that they allow an agent-based approach [94], and provide a means of comparing system parameters that otherwise may be empirically impossible or unethical to ascertain [95]. Our study presents relative measures of the dispersal success of organisms characterized by different biological traits across a series of varying environmental contexts. Landscapes in our model were defined by fundamental attributes shared with the real world. Representing individual-specific ecological and behavioural traits in quasi-realistic landscapes provides information from model outcomes that is translatable to natural systems, as has been achieved with generic simulations such as FRAGGLE and PATH [96,97]. In our study we aimed to explore a broad parameter space, so that we could identify consequences for dispersing under increasing landscape fragmentation that may be generalizable to other systems, and provide a basis for more specific studies within these systems. Previously, models of dispersal processes often have assumed static (i.e. invariant) environments. The need to understand dispersal behaviour in both spatially and temporally heterogeneous landscapes is now well recognized [29,98,99]. Studies that have assessed the effects of temporal change in landscape structure and composition on dispersal success suggest that increasing habitat fragmentation is less detrimental to organisms that disperse more frequently, and also, encourages development of multiple dispersal strategies within species [100,101]. This latter finding, together with the results of our study, prompts asking several questions of particular relevance to future empirical research: (i) how is dispersal behaviour mechanistically dependent on spatial and temporal context for different species?; and (ii) how do interactions between different dispersal syndromes and environmental composition/structure drive the evolution of dispersal-related traits [22,102]?

## 5. Conclusion

Our study presents new evidence of the need for context-specific characterizations of dispersal processes that realistically reflect the influence of biological attributes, as well as environmental structure [3]. We conclude that, both when assessed independently or integrated with population models, patterns of animal dispersal are best represented in simulation models as the consequence of fixed and condition-dependent organismal traits that interact with environmental attributes. Although such representations undoubtedly increase model complexity, inclusion of greater structural realism that reflects the multifarious determinants of dispersal success in most cases will enhance the heuristic value of dispersal models [102]. Knowledge of how animals interact with their environments is likely to be especially important for advancing understanding of how species differentially respond to anthropogenic perturbations, especially degradation and/or loss of areas that are vital to preservation of biodiversity, as well as their responses to global climate change.

## Abbreviations

MINAREA: minimum required area of habitat patches; HABAMT: habitat amount, total within landscape; HABAGG: spatial aggregation of habitat patches; BRT: Boosted regression tree; LHS: Latin hypercube sampling; SEABM: Spatially explicit, agent-based model/modelling; ODD: Overview, Design concepts and Details.

## Supplementary Material

Overview, Design Concepts, Details and Preliminary testing and parameterisation of the dispersal model

## Supplementary Material

Statistical methodology

## Supplementary Material

Model verification via independent analysis of each biological trait and its interactions with landscape structure

## Data accessibility

Data used in this study and model code are available on Dryad digital repository at: http://dx.doi.org/10.5061/dryad.rp8mp68
