# Supplementary material for: Effects of mis-alignment between dispersal traits and landscape structure on dispersal success in fragmented landscapes
Source: R Soc Open Sci. 2019 Jan 16;6(1):181702. doi: 10.1098/rsos.181702 (PMC6366165; doi:10.1098/rsos.181702)
Supplement: Overview, Design Concepts, Details and Preliminary testing and parameterisation of the dispersal model [file rsos181702supp1.docx]

Atkins JL, Perry GLW, Dennis, TE. 2018 Effects of mis-alignment between dispersal traits and landscape structure on dispersal success in fragmented landscapes. *R. Soc. open sci.* **5**: 181702. (doi:10/1098/rsos.181702)

**Supplementary Material 1 Overview, Design concepts and Details**

The Overview, Design concepts and Details (ODD) protocol [1,2] is a widely used framework for description and communication of individual-based models; our model description follows this structure.

**Purpose**

The purpose of our model was to evaluate how an organism’s fundamental biological characteristics influence dispersal success between isolated patches of suitable habitat in a fragmented landscape. Dispersal success was measured as the proportion of individuals in model runs that successfully reached a habitat patch other than where they started dispersing within a given time (250 model steps).

**Entities, State Variables, Scales**

We developed a spatially explicit, individual-based simulation, in which the elements of study are landscapes, habitat patches (constructed of groups of grid cells) and individuals. Landscapes were discrete-space grids of 100 × 100 cells, with each cell representing c. 50 × 50 metres, resulting in an area extent of c. 5 × 5 km. Simulated landscapes were continuous mosaics of patches of habitat biologically ‘suitable’ for dispersing agents and non-habitat ‘matrix’ that had varying levels of resource ‘quality’ (representing available food). The proportional abundance and configuration (aggregation) of the habitat patches were set by the ‘proportion-suitable’ and ‘attract-suitable’ parameters, respectively (Table A1.1). To represent different levels of habitat fragmentation, the proportion of suitable habitat was varied from low (0.05) to high (0.50) values, over 10 equal levels, and the strength of habitat aggregation (which determined the number and the size of the habitat patches) was varied over four levels, from randomly distributed (0.0) to highly aggregated (0.75) landscapes (Figure A1.1; Table A1.1).

Table A1.1 Parameter values for the dispersal simulation model.

| **Parameter/variable** | **Value/range of values** |
| --- | --- |
| **Landscape** |  |
| Total landscape size | 100 × 100 grid cells |
| Representative size of each grid cell | 50 × 50 metres |
| Quality value of a patch | 0.0-1.0 |
| Quality percentile for ‘forage’ patches | Highest 15% |
| Habitat amount (as proportion of total landscape) | 0.05-0.50 |
| Habitat attraction (aggregation among patches) | 0.0-0.75 |
| **Individual traits** |  |
| Speed of movement | 1-5 grid cells per-step |
| Mortality probability, per model step | 0.0001, 0.001, 0.005, 0.01, 0.015 |
| Perceptual range | 1-5 grid cells |
| Minimum habitat-area required | 1-50 grid cells |
| Foraging tendency (probability) | 0.0-0.5 |
| Maximum turning angle of correlated random walk | 90º |
| Angle of perceptual range | 180º |
| Energy gained from foraging | 1 step |
| Foraging speed | 0.1 grid cells per-step |
| **Population** |  |
| Initial number of individuals initialised | 2500 |
| **Temporal scale** |  |
| Maximum number of steps (stopping point) | 250 |
| Length of each time step | Approx. 1 minute (implied) |


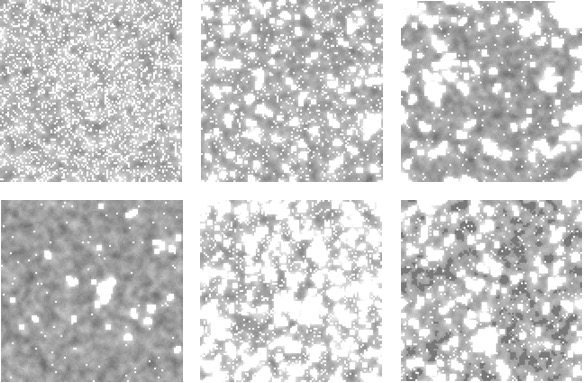


**Figure A1.1 Examples of simulated landscapes showing different levels of habitat availability and aggregation.** White patches are suitable habitat and grey areas represent the matrix; the grey-scaling represents the ‘foraging’ quality of each matrix cell, with darker shades representing higher foraging quality. Clockwise from the top left, landscapes parameterised by the following values: i) habitat amount of 25%, aggregation of 0; ii) habitat amount of 25%, aggregation of 0.25; iii) habitat amount of 25%, aggregation of 0.75; iv) habitat amount of 5%, aggregation of 0.5; v) habitat amount of 50%, aggregation of 0.5. The bottom right image shows a landscape under ‘baseline’ conditions (habitat amount of 25% and patch aggregation value of 0.5) with an example of allocated foraging patches (shown in darker grey).

Each non-habitat matrix grid cell was characterised by a resource ‘quality’ value, which represented the level of food available in that cell. We used a local averaging algorithm (see [3]) to generate a heterogeneous surface that varied in the quality of areas available for foraging (Figure A1.2). This method involved seeding all cells in the landscape matrix with a random uniform deviate between zero and one, and then applying local averaging at the level desired (note that local smoothing preserves the total amount of food resource available but allocates it more evenly across the grid). Clusters of matrix cells that had resource-quality values in the upper 15% of all grid cells in the landscape were deemed ‘foraging’ patches, in which individual agents searched for food resources based on their parameter-dependent tendency to do so.


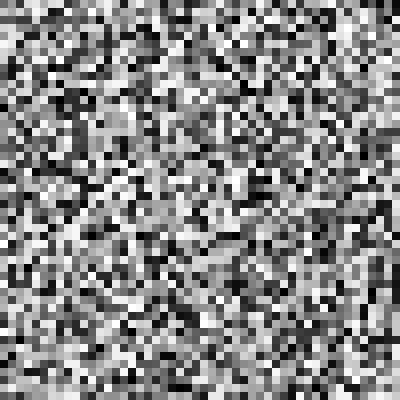

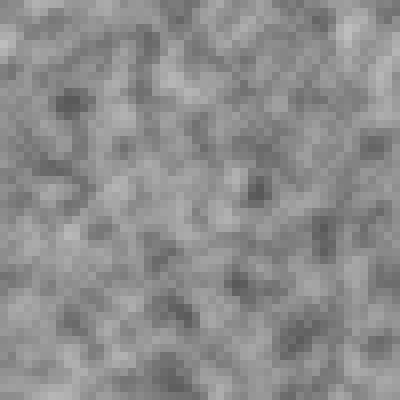

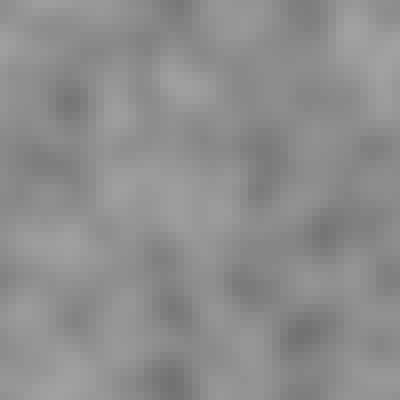


Figure A1.2 Example of the process of local averaging in a landscape. Patches were averaged using a weighted value of 0.1; each of the higher-valued dark patches shared 0.1 of their value between their eight neighbouring grid cells. During each time step, all neighbouring patches increased their value by 0.0125 (0.1 / 8), and dark patches proportionally decreased their values by 0.1. From left to right, images were taken at time-steps t = 0, t = 20, t = 50. Images from a model by O’Sullivan & Perry [3]developed in NetLogo [4].

Individuals were characterised by state variables related to intrinsic biological traits (Table A1.2). The time-step in the model was not explicitly defined, but given the spatial grain, each tick can be considered to represent about one minute in real time, constituting a typical movement speed during dispersal of 3 km/hr at a 50-m grid-cell resolution.

Table A1.2 State variables for dispersing individuals in the simulation model.

| **State variable** | **Description** |
| --- | --- |

| Start habitat  Current habitat  Total steps  Movement mode  **Biological traits**  Movement speed  Perceptual range  Mortality probability  Minimum patch area required  Foraging tendency | Habitat patch where the individual was randomly initialised  Individual current location (grid ells)  The number of steps (grid cells) an individual had taken at any point in time  The ‘behavioural’ mode in which an individual moved: either foraging or dispersing  Speed of movement (measured in grid cells per step)  Distance from current location that an individual could ‘sense’ the landscape (measured in grid cells)  Per time-step probability of mortality  Minimum area (number of grid cells) required by an individual for a habitat patch to be suitable for settlement  Probability an agent entered foraging mode when a foraging-patch was encountered |
| --- | --- |

**Process Overview and Scheduling**

Figure A1.3 provides a schematic representation of the model schedule. First, a landscape was generated, individuals then were initialised sequentially (there were no inter-individual interactions) and randomly placed at the edge of one habitat patch. Individuals moved through the landscape until either they dispersed successfully (found a new habitat patch) or ‘died’ (the probability of mortality at each step was determined by the per-step mortality rate) or the predetermined limit of time steps (250) was surpassed; this procedure continued until 2500 individuals had attempted to disperse.

Set up landscape (habitat patches and foraging matrix)

Die & create new individual

Y

N

Y

N

Create individual

Move

Submodels: *found-habitat*, *forage*, *disperse*

If 2500 individuals initialised = STOP

New habitat patch?

Record successful dispersal

Below max-steps and no mortality?

Record unsuccessful dispersal

Figure A1.3 Flowchart of the model schedule. Diamond-shaped boxes represent decisions made by the dispersing individuals, for which the outcome was either Y (yes) or N (no). First, a landscape was generated, then an agent was parameterised, processed through the submodels, recorded the outcome of its dispersal attempt, and then a new individual was generated and the procedure repeated. ‘Max-steps’ represents the maximum number of model steps available for an individual to successfully complete a dispersal attempt (fixed at 250). A model run ended once 2500 individuals had attempted to disperse.

**Design Concepts**

• Emergence – Dispersal success emerged from the model via an individual’s movements, driven by interactions between landscape structure and the organism’s biological traits.

• Sensing – Individuals could detect, over specified distances, the presence of nearby patches of suitable habitat. The distance over which individual agents had this capacity depended on the ‘perceptual-range’ parameter.

• Stochasticity – Most model parameters included stochastic variation – parameter values were drawn from probability distributions rather than being fixed single values, and were used in the model probabilistically.

• Observation – For evaluation of the model, dispersal behaviour (movements) and success of individuals were observed.

**Initialisation**

The model was initialised by placing an individual on the edge of a randomly selected habitat patch, in a landscape with a set proportion (amount) of habitat and degree of fragmentation, defined by the parameters of ‘habitat amount’ and ‘degree of aggregation’.

**Submodels**

1. Walk – individuals moved from their starting habitat patch via a correlated random walk (i.e., at a random bearing, but with a greater tendency to move forward rather than backward). If the agent detected another habitat patch within its perceptual range, it entered the found-habitat sub-model (Figure A1.4). Otherwise, individuals dispersed or foraged, depending on their current mode of behaviour.

By default, individuals were in ‘dispersal’ mode and only occasionally and probabilistically switched to ‘foraging’ mode. Agents remained in ‘foraging’ mode until a random uniform deviate was less than or equal to the probability of ending their foraging bout (‘p-end’), in which case they reverted to ‘dispersal’ mode (Figure A1.4). This test was made at every time step. Differences between ‘p-start’ and ‘p-end’ values always were the same (0.15), with ­p-end < p-start. During each time-step, agents updated the identity of their current habitat patch, incremented the total number of steps they had moved according to their speed, and assessed whether they had arrived in a new habitat patch, and thus had dispersed successfully.

2. Found-habitat – individuals turned toward patches of suitable habitat that were within their perceptual range (Figure A1.5 (a)), after which they moved directly to one of these patches (selected at random if more than one were detected) at a rate dependent on their speed of movement parameter. Once in found-habitat mode, individuals no longer detected other habitat patches but could suffer background mortality.

3. Dispersal – individuals moved through the landscape matrix via a correlated random walk (maximum turning angle ± 90°), according to the ‘movement speed’ parameter (Figure A1.5 (b)).

4. Forage – individuals moved through foraging-patches via correlated random walks, at a rate determined by their foraging-speeds (set at a constant 0.1 cells per-step, irrespective of movement speed) – this parameter represented a ‘foraging’ cost (Figure A1.5 (c)). For each time-step spent in foraging mode, individuals gained steps (following parameter ‘foraging energy’), representing energetic benefits from foraging. Resource levels in each foraging patch did not change.

Regardless of the particular submodel an individual was in, at each time step there was a probability of mortality as set by the per-step mortality rate. A random number between 0 and 1 was generated and if this was greater than the per-step mortality rate set for that model run, the individual died.

t = 0?

N

Y

N

Suitable habitat patch in range?

Enter *found-habitat* submodel

Search for habitat patches in perceptual range

Y

N

Mode ‘foraging’ & random ≤ *p*-end?

N

Y

Mode ‘dispersal’ & random ≤ *p*-start?

In foraging-patch?

N

Y

Set mode ‘foraging’, enter *forage* submodel

Set mode ‘dispersal’, enter *dispersal* submodel

Set mode ‘dispersal’, enter *dispersal* submodel

Enter *forage* submodel

N

Y

In matrix or start habitat patch?

Stop

Successful dispersal

N

Y

No mortality & steps ≤ max-steps?

Stop –

Unsuccessful dispersal

Y

Figure A1.4 Flow diagram of the main *walk* sub-model that determined how each individual moved through its landscape in the simulation model. ‘Max-steps’ is the maximum number of steps permitted for an individual to complete its dispersal attempt (set at 250). ‘*P*-start’ is the probability an individual entered ‘foraging’ mode when a foraging-patch was encountered during dispersal. ‘*P*-end’ is the probability of switching back to ‘dispersal’ mode from ‘foraging’ mode, while in a foraging-patch.

(a) (b) (c)

Face suitable habitat patch and move towards it at speed level

Increase total steps by speed level

Return to *walk* submodel

Move with correlated random walk at speed level

Increase total steps by speed level

Return to *walk* submodel

Move with correlated random walk at foraging speed level

Decrease total steps by foraging energy level

Return to *walk* submodel

Figure A1.5 Flow diagrams of the (a) *found-habitat*, (b) *dispersal* and (c) *foraging* submodels. Each diagram depicts the steps taken by an individual when it entered any particular submodel for one time-step.

**Preliminary testing and parameterisation of the dispersal model**

**Stopping point, number of replicates and parameter ranges**

The stopping point of our model was an elapsed time threshold at which dispersal was deemed to have been unsuccessful. This state was defined by the maximum number of steps that an individual could seek a habitat patch in which to settle (250 steps; Table A1.3).

The required number of replicates for each run of the model (i.e., the number of dispersers, Figure A1.6) and of each parameterisation of the model were determined through consistency analysis [5]; that is assessment of the model’s ‘natural’ variation (variation in the output of dispersal success with fixed parameter values, caused by underlying stochastic components of the model). We found that the mean and standard deviation of dispersal success did not change when we increased our sample size from 100 to 1000 model runs (mean = 0.8, standard deviation = 0.022). Based on these tests, 100 repetitions of each model parameterisation were conducted, each comprising 2500 individuals.

The range and intervals for both the environmental parameters (proportion/amount of habitat and degree of attraction among habitat patches) and individual trait values (speed, mortality rate, perceptual range, minimum habitat-patch-size and probability of foraging) were determined by running tests of the model in which parameter values were varied over their entire potential range at a variety of intervals. Figures A1.7-12 provide examples of parameters and graphical representation of this testing procedure.

Table A1.3 Equations used to calculate an appropriate stopping point for each run of the model, which was the maximum number of steps an individual had to find a new habitat patch until that dispersal attempt was recorded as a failure. The density of patches used was for the most fragmented landscape, where only 5% of the analytical domain was comprised of suitable habitat and maximum aggregation, because these were the conditions under which the nearest neighbour distances between patches were greatest. As distance travelled in a random walk scales to the square root of time, travelling nine units of distance should on average require 81 steps. Therefore, a maximum number of steps of 250 (as set in the model) allowed ample time for the agents to reach a new habitat patch.

| **Factor** | **Equation** | **Outcome** |
| --- | --- | --- |
| Expected lifetime (mean) | = Duration of a step (ticks) / per-step mortality rate  = 1 / 0.005 | 200 |
| Mean nearest-neighbour distance | = ½ √*D*  *D* = Density of patches (landscape area/ number of habitat patches)  = 0.5 * √(10201/30) | 9 |
| Distance travelled per tick | = α √*t*  *t* = 1/9  = α √1/9 | 0.11^·^ |
| Time to travel to nearest neighbour | = mean distance / distance travelled  = 9 / 0.11^·^ | 81 steps (under a simple random walk) |


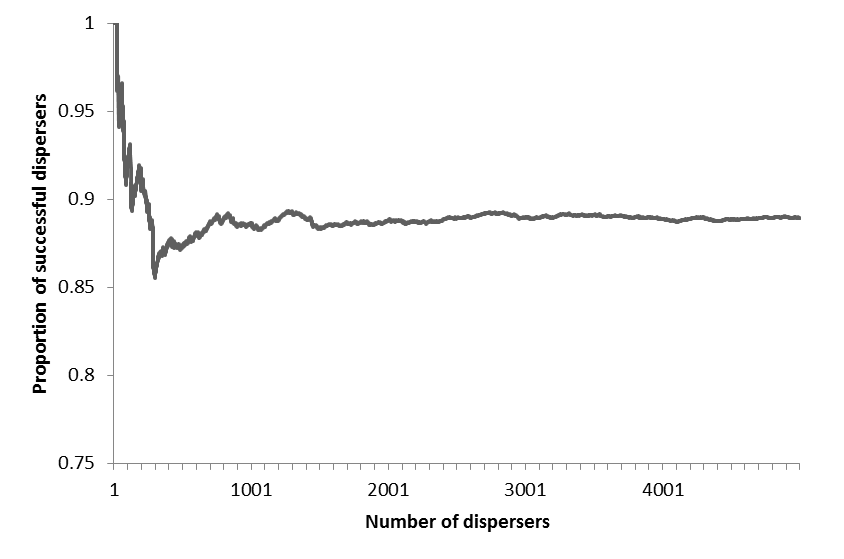


**Figure A1.6 Results of test runs of the dispersal model to decide on the number of replicates required for each run of the model, i.e., the number of dispersing agents for each model run.** The figure shows the number of successful dispersers as a proportion of the total number of dispersers released. The proportion of successful dispersers stabilised around 2000 evaluated agents. Therefore, the model was run with 2500 agents for each simulation to ensure the dispersal success reflected the average dispersal success in a particular landscape configuration.


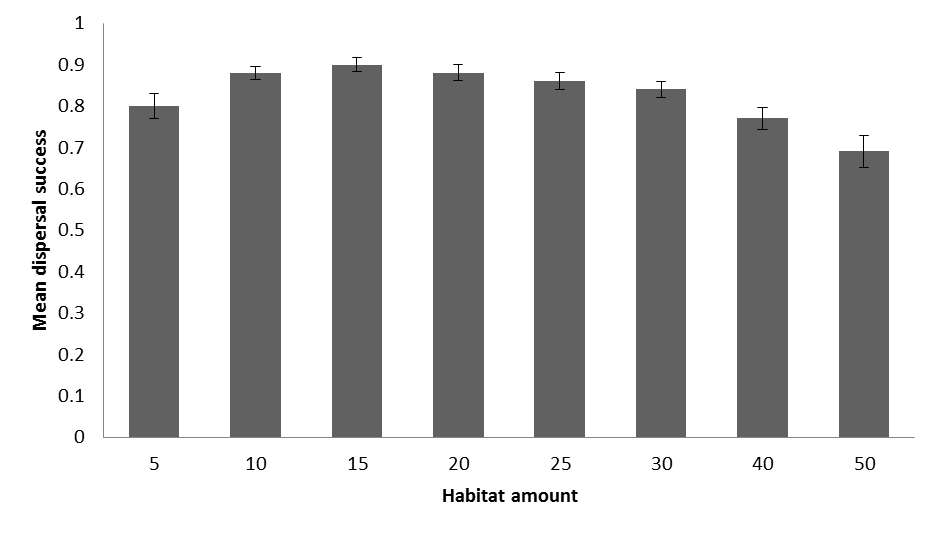


**Figure A1.7 Mean dispersal success when proportion (‘amount’, expressed as a percentage of the simulated landscape) of suitable habitat varied between 5 and 50% of the total area of the landscape, under baseline settings.** Error bars represent ± 1 standard deviation. Dispersal success was sensitive to small changes in the amount of suitable habitat in the simulated landscapes. Therefore, the proportion of suitable habitat was varied at 5% intervals and ranged from 5% to 50%.


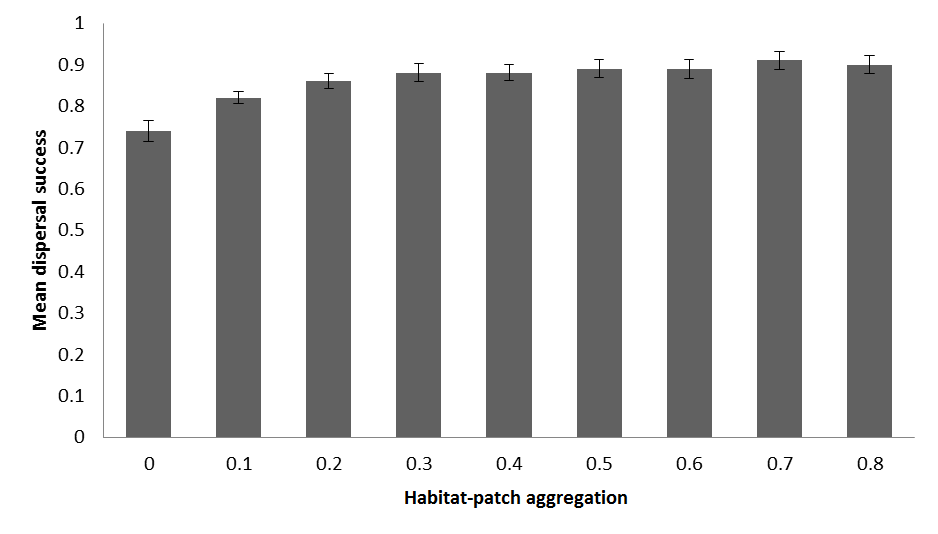
**Figure A1.8 Mean dispersal success when aggregation habitat patches in the simulated landscapes varied between 0.0 and 0.8, under baseline settings.** Error bars represent ± 1 standard deviation. Dispersal success increased with increasing spatial aggregation of patches but did not show strong sensitivity to small increases. On this basis, parameter values were set to vary between 0.0-0.75, at 0.25 intervals.


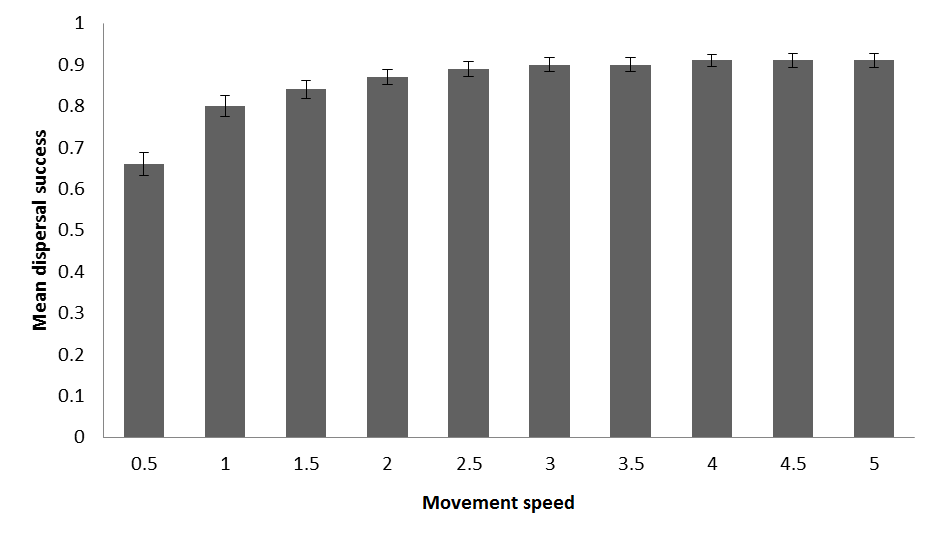


**Figure A1.9 Mean dispersal success of model agents in relation to speed of movement, varying between 0.5 and 5 NetLogo grid cells per-step, under baseline settings.** Error bars represent ± 1 standard deviation. Dispersal success was sensitive to differences movement speed when speeds were low. Therefore, in the model speeds were set to vary from 1-5 grid cells, at intervals of 1 grid cell.


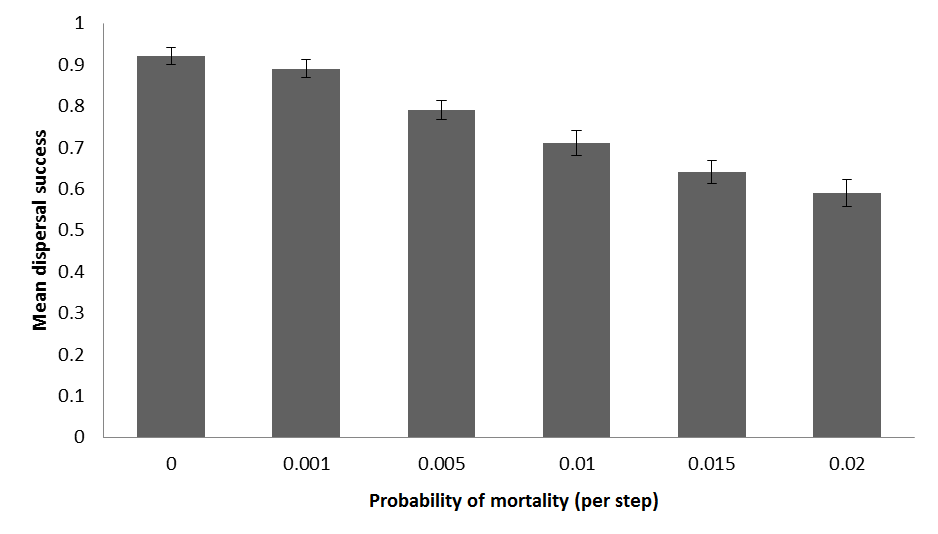


**Figure A1.10 Mean dispersal success in relation to increasing mortality rate (per step), over values of 0.0 to 0.02, under baseline settings.** Error bars represent ± 1 standard deviations. Dispersal success decreased with increasing mortality rate; it did show a high level of sensitivity between the values of 0 to 0.15. Therefore, values in the final model were set at 0, 0.001, 0.005, 0.01 and 0.


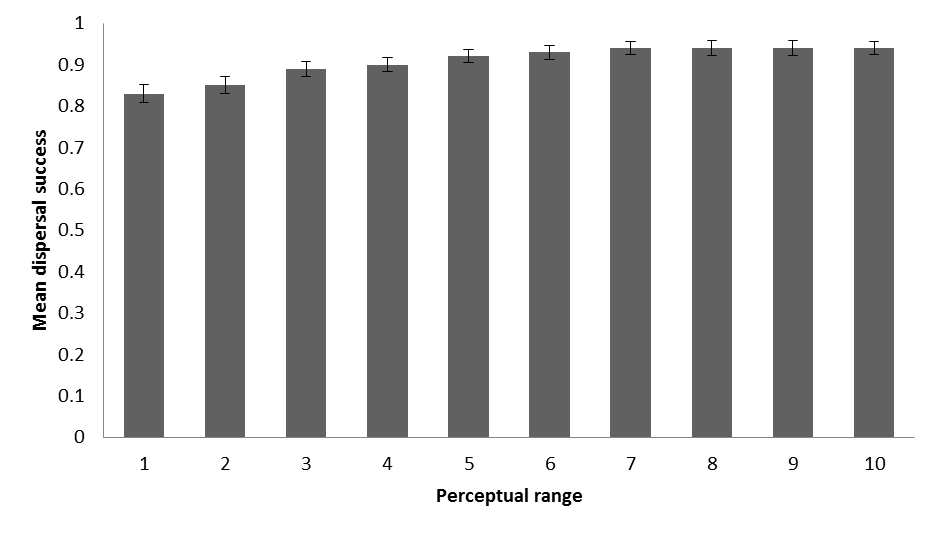


**Figure A1.11 Mean dispersal success in relation to variation in perceptual range from 1 to 10 landscape grid cells, under baseline settings.** Error bars represent ± 1 standard deviations. Dispersal success increased with increasing perceptual range, with a high level of sensitivity between 0 to 5 cells, but much lower sensitivity over higher perceptual ranges. Accordingly, the model values of perceptual range varied between 1 to 5 cells, at intervals of 1 cell.


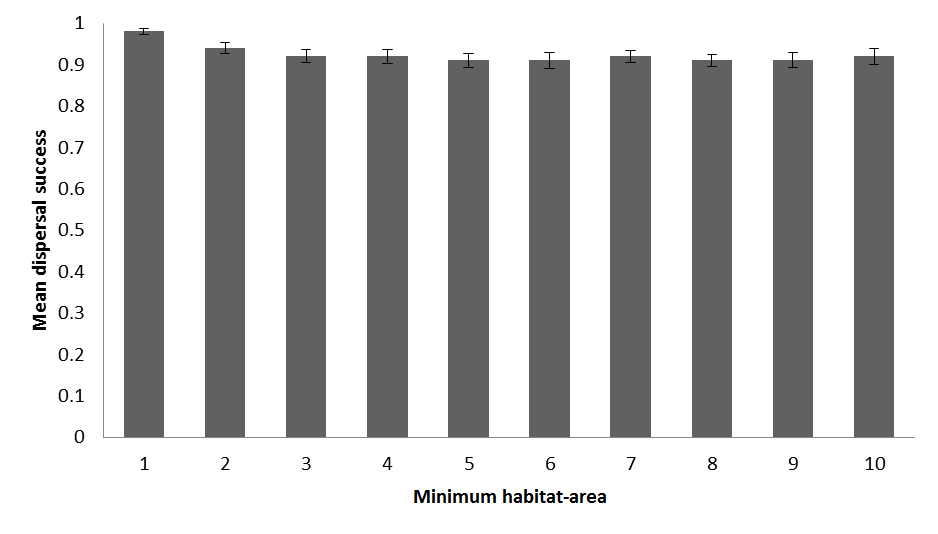


**Figure A1.12 Mean dispersal success in relation to minimum habitat-patch-size, from 1 to 10 NetLogo landscape grid cells, under baseline settings.** Error bars represent ± 1 standard deviations. Dispersal success decreased with increasing habitat-patch-size, but the differences were small. In the model this parameter was set to vary between 1 and 50 cells.

Range values for the foraging-tendency parameter were determined by the need to: i) simulate scenarios in which no foraging occurred; ii) limit the probability of foraging during a model step at 50% (max=0.5); and iii) standardise the difference between the probability of starting and ending foraging bouts (set at 0.15), so that the difference was equivalent across all foraging-tendency values. This constraint resulted in forging-tendency values of 0, 0.2, 0.3, 0.4 and 0.5.

**References**

1. Grimm V, Berger U, DeAngelis DL, Polhill JG, Giske J, Railsback SF. 2010 The ODD protocol: A review and first update. *Ecol. Modell.* **221**, 2760–2768. (doi:10.1016/j.ecolmodel.2010.08.019)

2. Grimm V *et al.* 2006 A standard protocol for describing individual-based and agent-based models. *Ecol. Modell.* **198**, 115–126. (doi:10.1016/j.ecolmodel.2006.04.023)

3. O’Sullivan D, Perry G. 2013 *Spatial Simulation: Exploring Pattern and Process*. Chichester, U.K.: John Wiley & Sons, Ltd.

4. Wilensky U. 1999 NetLogo. Center for Connected Learning and Computer-Based Modeling.

5. Grimm V, Railsback SF. 2005 *Individual-based Modeling and Ecology*. Princeton: Princeton University Press.
