# Supplementary material for: Effects of mis-alignment between dispersal traits and landscape structure on dispersal success in fragmented landscapes
Source: R Soc Open Sci. 2019 Jan 16;6(1):181702. doi: 10.1098/rsos.181702 (PMC6366165; doi:10.1098/rsos.181702)
Supplement: Statistical methodology [file rsos181702supp2.docx]

Atkins JL, Perry GLW, Dennis, TE. 2018 Effects of mis-alignment between dispersal traits and landscape structure on dispersal success in fragmented landscapes. *R. Soc. open sci.* **5**: 181702. (doi:10/1098/rsos.181702)

**Supplementary Material 2 Statistical Methodology**

**Latin hypercube sampling (LHS)**

This technique is a form of uncertainty analysis in which the full range of potential values for uncertain parameters are explored through generation of a representative sample of all possible combinations of parameter values from distributions with multiple parameters (for detailed explanation of this sampling process, see [1]).

LHS was performed in the statistical computing environment R, using the RNetLogo package [2] that was designed so that NetLogo models could be called from R and analysed using R functions, such as LHS. In procedure, 10,000 runs of the model were conducted and dispersal success was measured for each model implementation; each run represented an independent sample of the parameter space, the ranges for which are shown in Table 1. R data-frames were generated that contained the parameter values used for each of the 10,000 model runs, and dispersal success was determined for each run.

| **Parameter** | | **Range** | **Discrete or continuous?** | | |
| --- | --- | --- | --- | --- | --- |
| **Landscape** |  | | |  | |
| *Habitat amount* | | 5-50% | Continuous | |  |
| *Habitat attraction* | | 0-0.75 | Continuous | |  |
| **Individual traits** | |  |  | |  |
| *Speed* | | 1-5 | Discrete | |  |
| *Mortality probability, per-step* | | 0.0001-0.01 | Continuous | |  |
| *Perceptual range* | | 1-5 | Discrete | |  |
| *Minimum habitat-patch size* | | 1-50 | Continuous | |  |
| *Foraging probability (p-start)* | | 0-0.5 | Continuous | |  |

Table 1 Ranges of parameter values used for Latin hypercube sampling of the complete model-parameter space

**Broken-stick random number generation**

Broken-stick statistical distributions typically are used to describe ecological community structure (e.g., species abundance relationships), but in their simplest form are one-dimensional vectors partitioned into *n* segments by *n* – 1 random divisions [3]. In the trade-off analysis of our study, ‘sticks’ were 100 units long (representing the total level of allocation to traits available) and the number of units in each segment represented the allocation to each biological trait. For every point allocated to a trait, that trait increased or decreased from its baseline parameter value by a specific factor (1% of the parameter value range; Table 2). Segments were randomly generated and each trait then randomly assigned a segment of points, which determined the trait value for that simulation.

Table 2 Parameter-value ranges and specialisation in biological traits for trade-off analysis of the dispersal model. Values of each parameter started at base quantities and increased or decreased by an amount that was equal to the number of points allocated to that trait multiplied by a given coefficient (this scales the increase/decreases to the total available units of 100).

| **Trait** | **Range** | **Base** | **Multiplier** | **Increase or decrease?** |
| --- | --- | --- | --- | --- |
| *Speed* | 1-5 | 1 | 0.04 | + |
| *Perceptual range* | 1-5 | 1 | 0.04 | + |
| *Mortality probability* | 0.0001-0.01 | 0.01 | 0.000099 | - |
| *Foraging tendency* | 0-0.5 | 0 | 0.005 | + |
| *Minimum habitat-patch size* | 1-50 | 50 | 0.49 | - |

**References**

1. Helton JC, Davis FJ: **Latin hypercube sampling and the propagation of uncertainty in analyses of complex systems.** *Reliab Eng Sys Safe* 2003, **81:**23–69.

2. Thiele JC, Kurth W, Grimm V: **RNETLOGO: an R package for running and exploring individual-based models implemented in NetLogo**. *Method Ecol Evol* 2012*,* **3:**480–483.

3. Wilson JB: **Would we recognize a broken-stick community if we found one ?** *Oikos* 1993, **67:**181–183.
