# Supplementary material for: Effects of mis-alignment between dispersal traits and landscape structure on dispersal success in fragmented landscapes
Source: R Soc Open Sci. 2019 Jan 16;6(1):181702. doi: 10.1098/rsos.181702 (PMC6366165; doi:10.1098/rsos.181702)
Supplement: Model verification via independent analysis of each biological trait and its interactions with landscape structure [file rsos181702supp3.docx]

Atkins JL, Perry GLW, Dennis, TE. 2018 Effects of mis-alignment between dispersal traits and landscape structure on dispersal success in fragmented landscapes. *R. Soc. open sci.* **5**: 181702. (doi:10/1098/rsos.181702)

**Supplementary Material 3 Sensitivity Analysis**

The effect of each biological trait on dispersal success was depicted graphically. Figures 1-5 show the median dispersal success for each trait-parameter value from 100 model repetitions, plotted against habitat amount, with each unit of attraction/aggregation among habitat patches compared in sub-plots (labelled a to d).

Across all levels of habitat attraction (i.e., as fragmentation decreases) and the range of parameter values for all biological traits, dispersal success was positively correlated with the amount of habitat in the landscape (Figures 1-5). Furthermore, when little habitat was available and the landscapes were highly fragmented (habitat amount and patch aggregation: 0.05-0.1, and 0, respectively), dispersal success was consistently low over all the parameter values for each biological trait. Increasing values of habitat aggregation represent more clustered landscapes and habitat amounts are presented as proportions of the total landscape area.

At higher levels of aggregation (Figure 1 (b), (c) and (d)), dispersal success increased markedly as movement speed increased, especially between 1 and 2 grid cells per step. This effect was much weaker when proportions of suitable habitat (‘amount’) were greater than 0.2). Differences in dispersal success between movement speeds of 1 and 2 grid cells per step increased with greater aggregation of habitat patches (Figure 1 (a)-(d)).


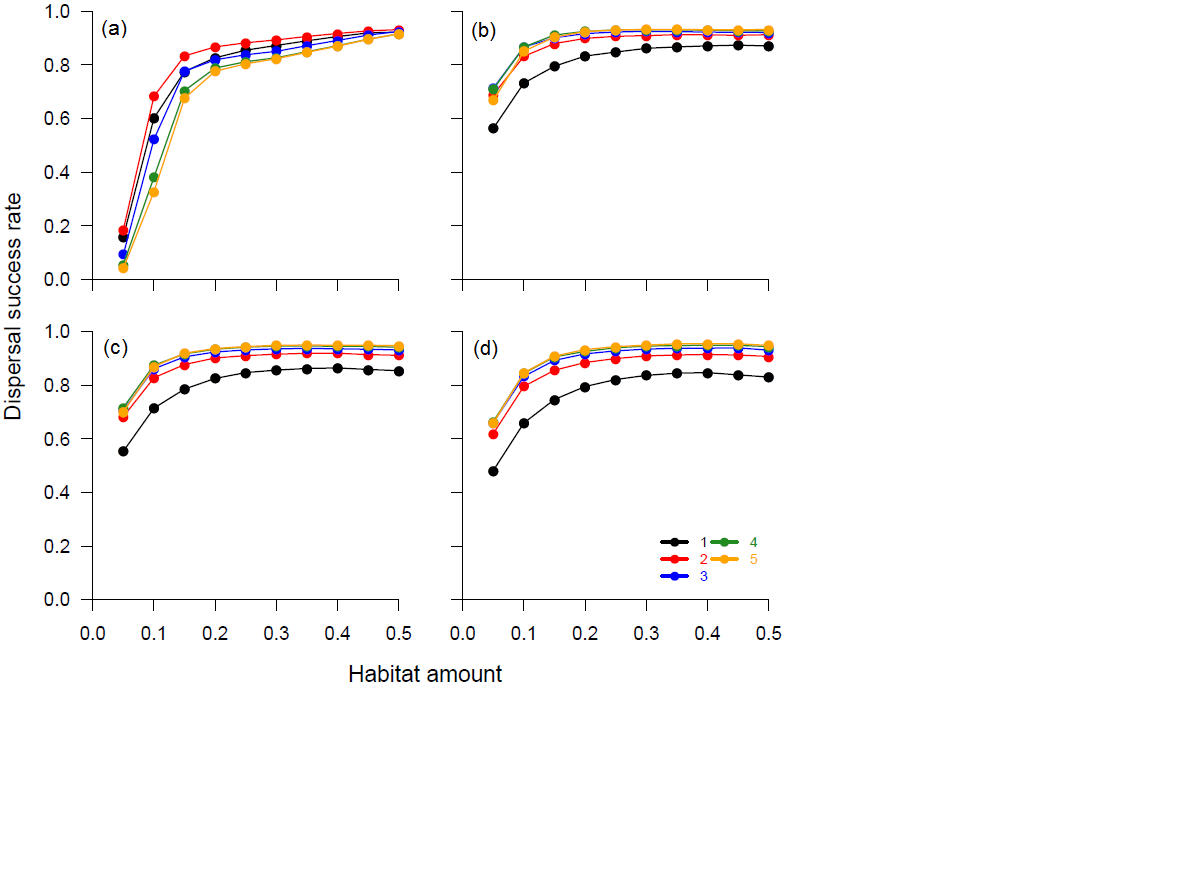


**Figure 1 Dispersal success of model agents in relation to different movement speeds, amounts of suitable habitat (‘HABAMT’), and levels of habitat aggregation (‘HABAGG’).** Movement speeds are represented by differently coloured lines, and are reported in the number of NetLogo cells per model step. Levels of habitat-patch aggregation are: 0 (a), 0.25 (b), 0.50 (c), and 0.75 (d). All other model parameters were fixed at baseline values (for values, see main text).

As the extent of habitat fragmentation fell (Figures 2 (b), (c) and (d)), perceptual range showed a slight positive correlation with dispersal success, but only when the amount of suitable habitat in the landscape was low (less than 0.2). The greatest advantage of an increased perceptual range occurred when habitat amount was low and aggregation among habitat patches was high (Figure 2 (c) and (d)).

*
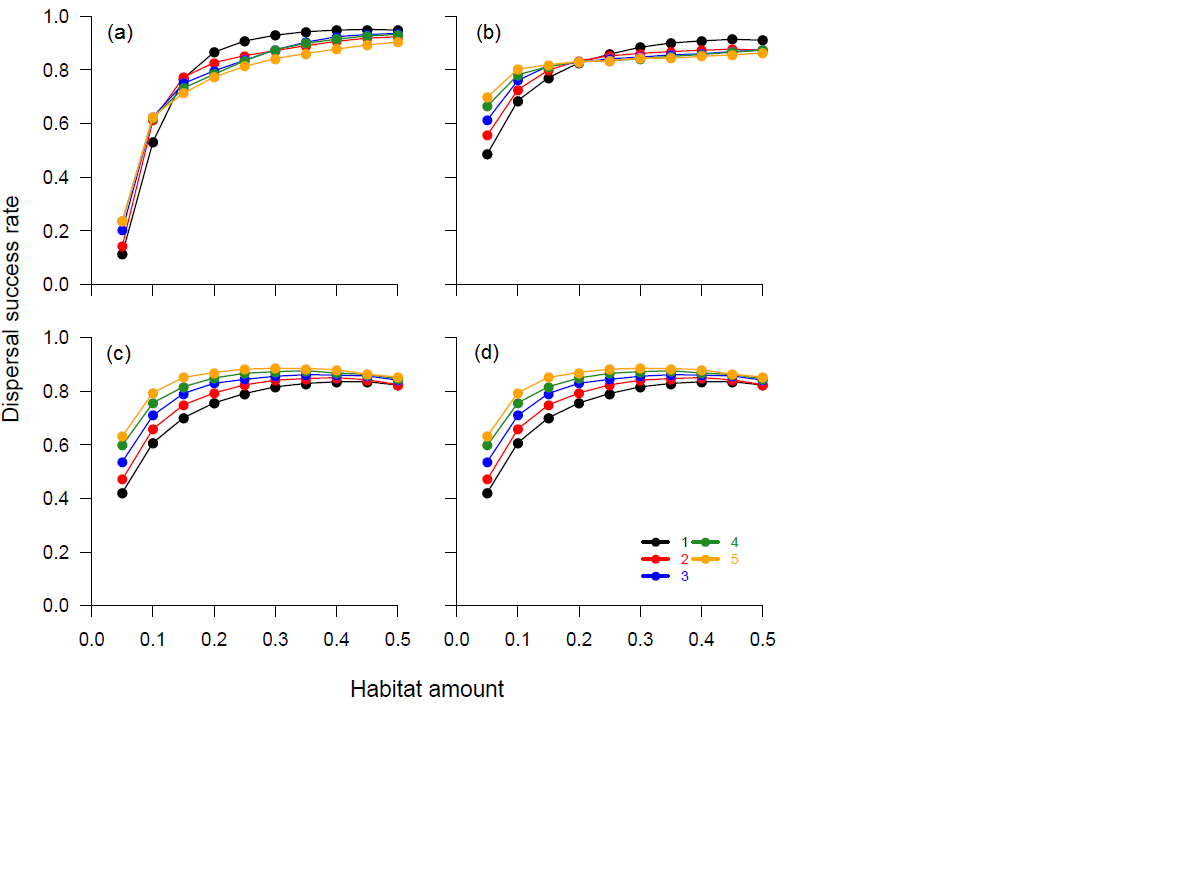
*

**Figure 2 Dispersal success of model agents in relation to different perceptual ranges, amounts of suitable habitat (‘HABAMT’), and levels of habitat aggregation (‘HABAGG’).** Perceptual ranges are represented by differently coloured lines, and are reported as the number of NetLogo cells the range spans around each agent. Levels of habitat-patch aggregation are: 0 (a), 0.25 (b), 0.50 (c), and 0.75 (d). All other model parameters were fixed at baseline values (for values, see main text).

At all levels of habitat aggregation, dispersal success showed a negative relationship with mortality probability (Figure 3 (a) - (d)). As the landscape became more aggregated (Figure 3 (c) and (d)), increased probability of mortality had a greater influence on dispersal success, even as the amount of habitat in the landscape increased. Dispersal success showed a slight decline at high levels of habitat amount and aggregation. This apparently count-intuitive outcome occurs because, in highly aggregated landscapes with large amounts of habitat, there are only a small number of separate habitat patches, therefore the chances of individuals finding new patches to successfully disperse to are decreased. Such decreases are not observed in other analyses of individual biological traits because these were run with the ‘baseline’ mortality susceptibility value (0.005).


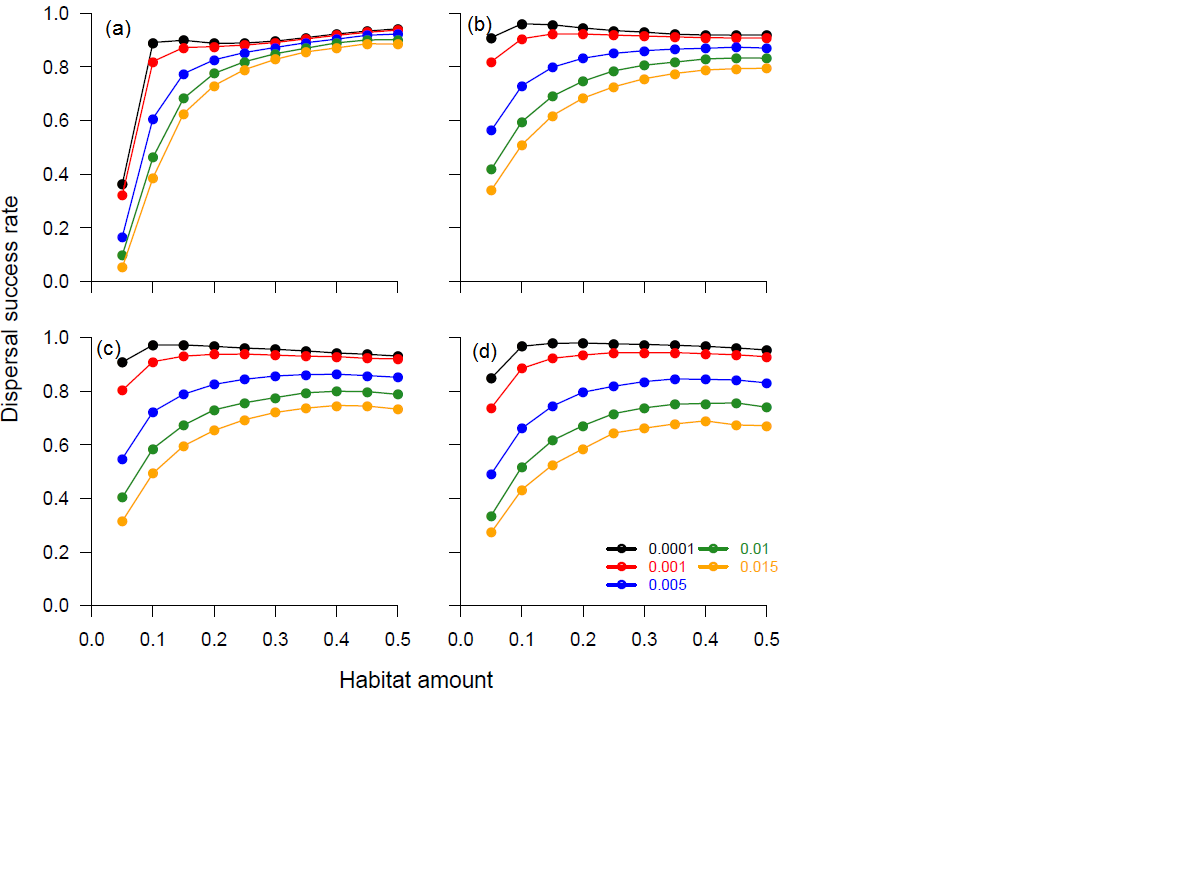


**Figure 3 Dispersal success of model agents in relation to different mortality probabilities, amounts of suitable habitat (‘HABAMT’), and levels of habitat aggregation (‘HABAGG’).** Probabilities of mortality are represented by differently coloured lines, and are reported as the probability of an individual dying due to extrinsic causes for each model step. Levels of habitat-patch aggregation are: 0 (a), 0.25 (b), 0.50 (c), and 0.75 (d). All other model parameters were fixed at baseline values (for values, see main text).

When proportions of suitable habitat and aggregation among habitat patches both were low, dispersal success was consistently poor, often being 0 for values of minimum habitat-patch-size requirement over one (Figure 4 (a)). Under these conditions, landscape consisted of only a few, small isolated patches, meaning that individuals with a minimum habitat-patch-size requirement of more than one were more likely to find a suitable habitat patch. In general, dispersal success was higher when minimum habitat-area required was lower (1–10). Differences in dispersal success for different levels of minimum habitat-area required decreased as habitat amount increased, at all aggregation levels (Figure 4).


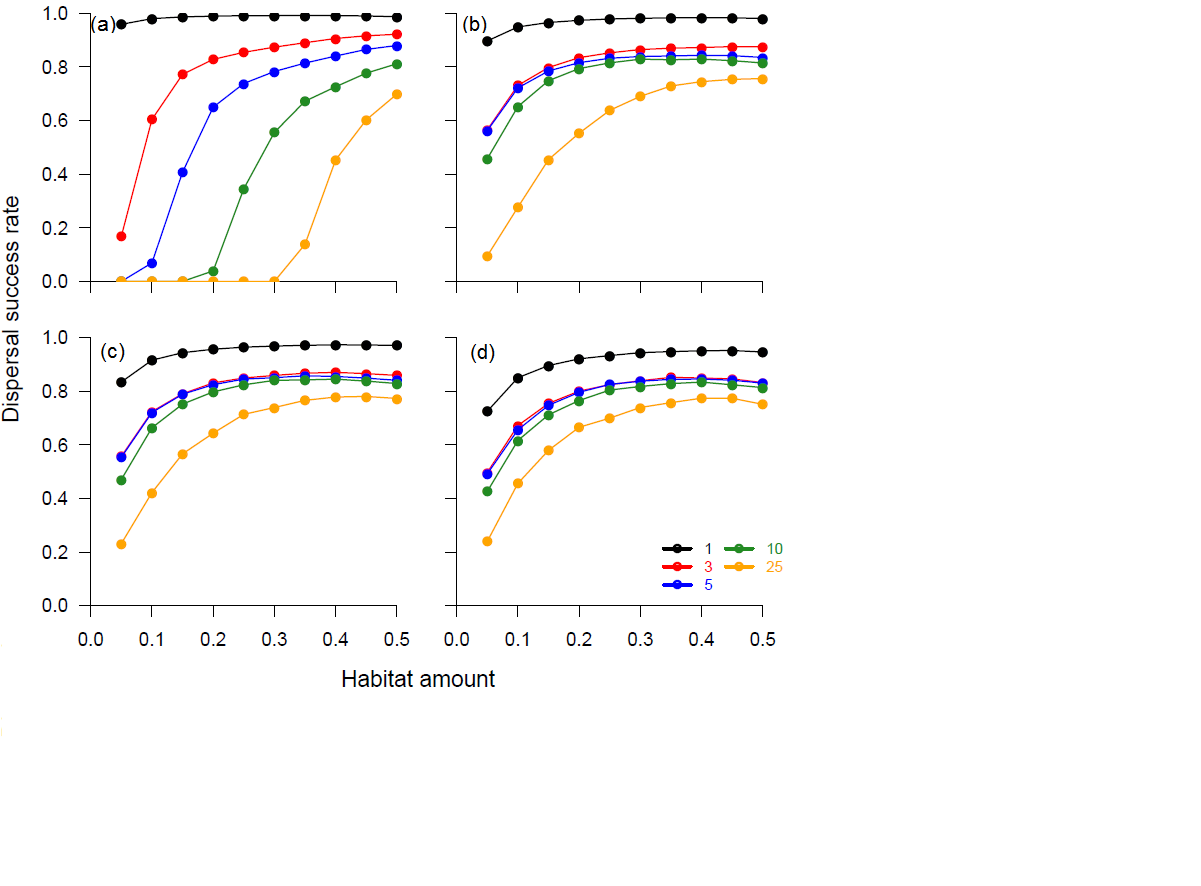


**Figure 4 Dispersal success of model agents in relation to different minimum habitat area requirements (‘MINAREA’), amounts of suitable habitat (‘HABAMT’), and levels of habitat aggregation (‘HABAGG’).** MINAREAs are represented by differently coloured lines, and are reported as the number of NetLogo cells per habitat patch. Levels of habitat-patch aggregation are: 0 (a), 0.25 (b), 0.50 (c), and 0.75 (d). All other model parameters were fixed at baseline values (for values, see main text).

Dispersal success essentially was independent of the probability of individuals switching to foraging activity during dispersal (Figure 5).


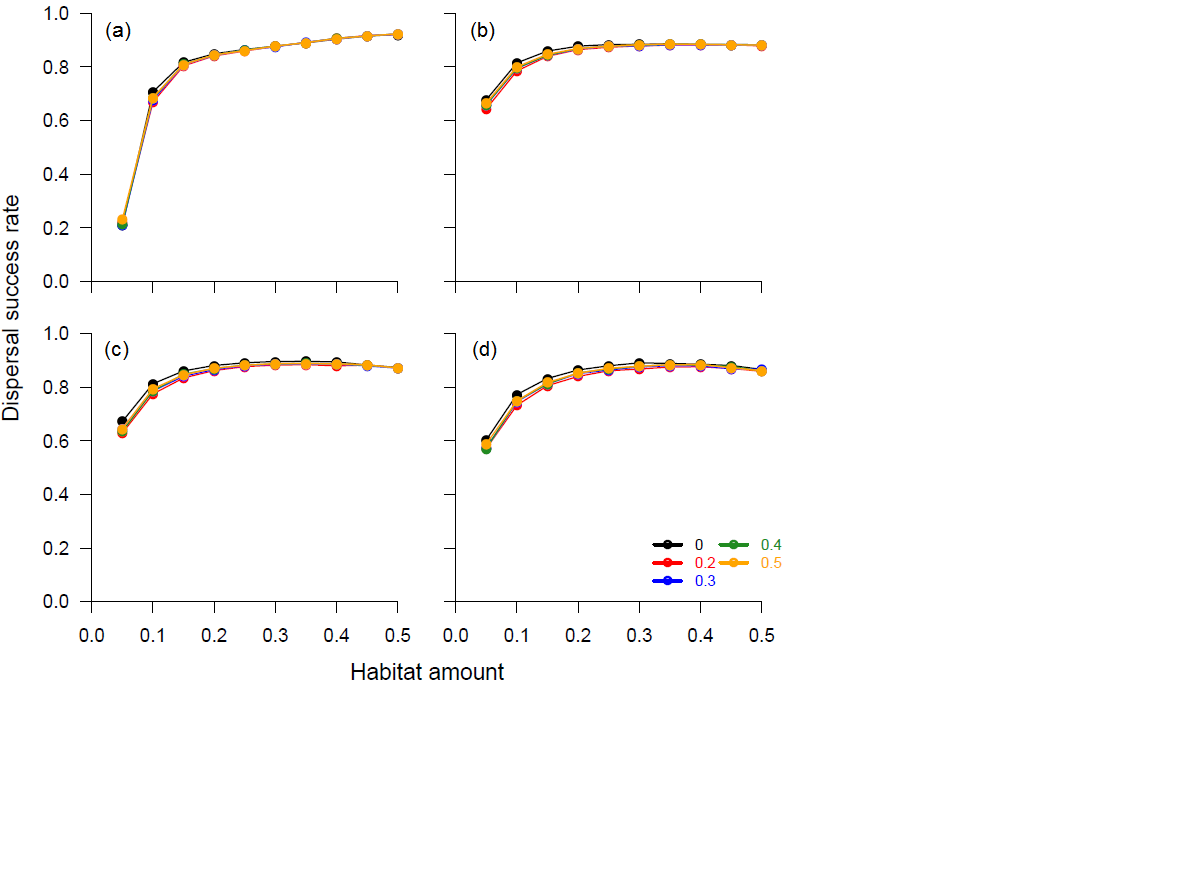


**Figure 5 Dispersal success of model agents in relation to different tendencies to forage during dispersal, amounts of suitable habitat (‘HABAMT’), and levels of habitat aggregation (‘HABAGG’).** Foraging tendencies are represented by differently coloured lines, and are reported as the probability of entering forage mode when in a foraging patch. Levels of habitat-patch aggregation are: 0 (a), 0.25 (b), 0.50 (c), and 0.75 (d). All other model parameters were fixed at baseline values (for values, see main text).
